# From ADHD to well-being: The Role of Rejection Sensitivity in college life

**DOI:** 10.1192/j.eurpsy.2024.594

**Published:** 2024-08-27

**Authors:** V. Müller, B. Pikó

**Affiliations:** ^1^Doctoral School of Education; ^2^Department of Behavioral Sciences, University of Szeged, Szeged, Hungary

## Abstract

**Introduction:**

Rejection-sensitivity is a prevalent yet understudied emotional symptom often associated with adult ADHD. While ADHD research typically focuses on behavioral and cognitive facets, emerging evidence highlights the significance of emotional symptoms. Emotional dysregulation in ADHD impacts psychological well-being and mental health. Our study examines how ADHD symptoms relate to rejection sensitivity, considering factors like resiliency, self-regulation, and overall well-being.

**Objectives:**

Our study seeks to establish a direct connection between ADHD scores and rejection sensitivity among college students. We also investigate the mediating role of well-being, creative executive efficiency, self-regulation, and resilience, while exploring the moderating role of savoring capacity.

**Methods:**

Between February and May of 2023, we conducted a cross-sectional study using an online questionnaire, gathering data from 304 Hungarian higher education students aged 18 to 35. The majority, 78.0%, were female, and 71.4% were full-time students. Most participants were pursuing a bachelor’s degree (56.6%), followed by undivided master’s (21.7%), doctoral studies (13.8%), and traditional master’s degrees (6.9%). We administered the Adult ADHD Self-Report Scale (ASRS-v.1.1), The Mental Health Test (MHT), and the Rejection Sensitivity Questionnaire (A-RSQ) for our research.

**Results:**

First, the ADHD scores were significantly associated with each mediator (well-being: β = -.343, p < .001; creative and executive efficiency: β = -.183, p < .01; self-regulation (β = -.230, p < .001; and resilience: β = -.321, p < .001). There was a direct effect of ADHD scores on rejection sensitivity scores (β = .466, p < .001). Finally, we also detected the indirect effects of ADHD scores on rejection sensitivity scores through the four mediators (β = .227, p < .001). Savoring capacity significantly moderated the relationship between ADHD and rejection sensitivity scores (β = -.244, p < .001).

**Conclusions:**

ADHD scores in our study population significantly correlate with well-being, creative and executive efficiency, self-regulation, and resilience. Furthermore, these scores directly influence rejection sensitivity, suggesting a heightened vulnerability to perceived rejection among those with higher ADHD scores. The indirect effects emphasize that the relationship between ADHD and rejection sensitivity is mediated by the aforementioned positive psychological constructs. This underscores the need for holistic interventions in ADHD populations, addressing not just core ADHD symptoms but also enhancing well-being, cognitive efficiency, self-regulation, and resilience to potentially mitigate rejection sensitivity.

**Disclosure of Interest:**

V. Müller Grant / Research support from: This project received funding from the New National Excellence Program under the Ministry for Culture and Innovation, sourced from the National Research, Development, and Innovation Fund, reference #ÚNKP-23-3-SZTE-66., B. Pikó: None Declared

